# Deltaproteobacterium Strain KaireiS1, a Mesophilic, Hydrogen-Oxidizing and Sulfate-Reducing Bacterium From an Inactive Deep-Sea Hydrothermal Chimney

**DOI:** 10.3389/fmicb.2021.686276

**Published:** 2021-09-22

**Authors:** Nicole Adam, Yuchen Han, Katja Laufer-Meiser, Rebecca Bährle, Ulrich Schwarz-Schampera, Axel Schippers, Mirjam Perner

**Affiliations:** ^1^GEOMAR and Molecular Biology of Microbial Consortia, GEOMAR Helmholtz Center for Ocean Research Kiel, Kiel, Germany; ^2^GEOMAR and Molecular Biology of Microbial Consortia, Biocenter Klein Flottbek, University of Hamburg, Hamburg, Germany; ^3^Microbiology and Biotechnology, Biocenter Klein Flottbek, University of Hamburg, Hamburg, Germany; ^4^Federal Institute for Geosciences and Natural Resources (BGR), Hannover, Germany; ^5^International Seabed Authority, Kingston, Jamaica

**Keywords:** Deltaproteobacteria, Desulfobulbaceae, hydrogen oxidation, sulfate reduction, hydrothermal deep-sea vent

## Abstract

A novel deltaproteobacterial, mesophilic, hydrogen-oxidizing, and sulfate-reducing bacterium (strain KaireiS1) was highly enriched from an inactive chimney located in the active zone of the Kairei hydrothermal vent field (Central Indian Ridge) in the Indian Ocean. Based on 16S rRNA gene analyses, strain KaireiS1 is the currently only cultured representative of a cluster of uncultured Deltaproteobacteria, positioned within the Desulfobulbaceae family, between the *Desulfobulbus* genus and the “Cable Bacteria.” A facultative autotrophic lifestyle of KaireiS1 is indicated by its growth in the absence of organic compounds, measurements of CO_2_-fixation rates, and activity measurements of carbon monoxide dehydrogenase, the key enzyme of the reductive Acetyl-CoA pathway. Apart from hydrogen, strain KaireiS1 can also use propionate, lactate, and pentadecane as electron donors. However, the highest cell numbers were reached when grown autotrophically with molecular hydrogen. Hydrogen uptake activity was found in membrane and soluble fractions of cell-free extracts and reached up to 2,981±129 nmol H_2_*min^−1^*mg^−1^ of partially purified protein. Commonly, autotrophic sulfate-reducing bacteria from the *Deltaproteobacteria* class, thriving in hydrothermal vent habitats are described as thermophiles. Given its physiological characteristics and specific isolation source, strain KaireiS1 demonstrates a previously unnoticed potential for microbial sulfate reduction by autotrophs taking place at moderate temperatures in hydrothermal vent fields.

## Introduction

The Kairei hydrothermal field is located in the Central Indian Ridge (Gamo et al., 2001; Hashimoto et al., 2001). After the field’s discovery in 2001, its geological background and biological communities were investigated (Gamo et al., 2001; Hashimoto et al., 2001; Gallant and Von Damm, 2006): Kairei fluids are characterized by low pH, relatively high concentrations of H_2_S and H_2_, and low CH_4_ levels (Van Dover et al., 2001). Consistent with the fluid’s high H_2_ concentrations, many H_2_-oxidizing bacteria have been isolated from the Kairei hydrothermal field (Van Dover et al., 2001; Takai et al., 2004a,b). In addition, the thermophilic methanogenic isolates and marker-gene based identification of sulfate-reducing bacteria have been described (Van Dover et al., 2001; Nakagawa et al., 2004; Takai et al., 2004c).

Analyses of microbial community compositions in inactive Kairei chimneys have demonstrated the presence of members from the Desulfobulbaceae family (e.g., 2.2% *Desulfobulbus* spp. in the chimney sample S1; Han et al., 2018). This family currently comprises eight validated genera and two candidate genera with *Desulfobulbus* representing the type genus (Kuever, 2014; Junghare and Schink, 2015; Trojan et al., 2016). So far, most isolates of the Desulfobulbaceae family (except for members of the candidate genera Electrothrix and Electronema and a few other species) have been described as heterotrophic, sulfate-reducing bacteria (SRB; Kuever, 2014; Kjeldsen et al., 2019). *Candidatus* Electrothrix and *Candidatus* Electronema form the group of Cable Bacteria that currently does not comprise axenic cultures. However, insights into their physiology could be gained by means of enrichment cultures, metagenomics, and incubation-experiments (Kjeldsen et al., 2019; Müller et al., 2020). These bacteria couple the oxidation of sulfide to the reduction of oxygen and nitrate *via* long-distance electron transport across centimeter long filaments, which can be found in marine and fresh-water sediments (Marzocchi et al., 2014; Kjeldsen et al., 2019). The type genus *Desulfobulbus* comprises 10 well-described isolates, which are Gram-negative, strictly anaerobic, and mesophilic heterotrophic SRB, isolated from diverse marine sediments and terrestrial fields and a human oral niche. Apart from organic electron donors like lactate, formate, or pyruvate, some species can use molecular hydrogen albeit only in the presence of acetate (Widdel and Pfennig, 1982; Samain et al., 1984; Lien et al., 1998; Sass et al., 2002; Suzuki et al., 2007; Sorokin et al., 2012; El Houari et al., 2017; Kharrat et al., 2017; Cross et al., 2018).

While sulfate reduction in marine sediments has been extensively studied (Kasten and Jørgensen, 2000), fewer studies have focused on the microbial sulfate reduction in hydrothermally influenced sediments and hydrothermal deposits, restricted to the analysis of sulfate reduction rates for heterotrophs, the phylogenetic analysis of associated microbial communities, and the isolation of thermophilic, autotrophic SRB (Elsgaard et al., 1994; Sievert and Kuever, 2000; Nakagawa et al., 2004; Frank et al., 2013, 2015). As expected for hydrothermally influenced environments exhibiting elevated temperatures, the incubation experiments demonstrated increased sulfate reduction rates at temperatures above 50°C and the dominance of thermophiles in the associated microbial sulfate reducing communities (Elsgaard et al., 1994; Frank et al., 2013, 2015).

Here, we present work on a new deltaproteobacterial highly enriched strain (KaireiS1=DSM 108158) from an uncultured lineage positioned between the *Desulfobulbus* genus and Cable Bacteria, within the Desulfobulbaceae family. It was enriched in co-culture with a *Sunxiuqinia* species (a member of *Bacteroidetes*) from an inactive chimney – in close proximity to the main active Kairei vent field. Its morphological, physiological, and phylogenetic characteristics are described here and its physiology, as studied, placed into an environmental context.

## Materials and Methods

### Isolation of Strain KaireiS1

During the INDEX2016 cruise, an inactive chimney structure (06ROV03-A) was collected in close proximity to an active venting site of the Kairei field (25.32°S and 70.04°E) at a water depth of 2,420m by means of the ROV VICTOR6000 (Ifremer) as previously described (Han et al., 2018). Immediately after sample recovery on board, small pieces of the massive sulfides from this inactive chimney were stored in organic-free MJ medium (artificial sea water) with the redox indicator resazurin (Hansen and Perner, 2015). These pre-enrichments, intended to bridge the gap until enrichments could be performed under different growth conditions in the home laboratory, were carried out in 100ml Duran glass bottles. The bottles were filled with 50ml MJ medium, sealed with silicone septa, and spiked with sterile argon (Air Liquide, Paris, France) in the headspace to reduce oxygen concentrations in the gas phase. The medium turned from blue to colorless after a 2-week incubation on board at 4°C, indicating increasingly reduced conditions over time. After transportation to the home laboratory, the culture resulting in the dominance of strain KaireiS1 was enriched in MJ medium under an H_2_:CO_2_ (80:20) atmosphere (Westfalen AG, Münster, Germany) at 25°C in the dark.

The total DNA of the enrichment was extracted, and a clone library harboring the corresponding 16S rRNA genes was constructed and sequenced as described previously (Perner et al., 2009).

In order to isolate the most abundant species (i.e., strain KaireiS1) from the enrichment culture, serial dilutions were performed in MJ medium and AL20 medium (organic medium), which is a slight modification from the L20 medium (Lien et al., 1998), namely 2mM sodium acetate was added instead of sodium propionate. The headspaces were filled with H_2_:CO_2_ (80:20) gas mixture. In each case, six parallel dilutions from 1:10 to 1:10^10^ were performed. The dilutions were then used to inoculate the cultures of 50ml with AL20 or MJ medium in butyl stopper-sealed serum bottles and incubated anaerobically at 28°C with a H_2_:CO_2_ (80:20) gas mixture. Again, clone libraries were constructed and sequenced, and the procedure was repeated until only one species was identified among 48 clones of a library.

### Phylogenetic Analysis and Purity Check of Strain KaireiS1

The full 16S rRNA gene sequence from strain KaireiS1 (1,462bp, deposited under GenBank accession number MH763813) was analyzed using nucleotide Blast.^1^ The phylogenetic tree of strain KaireiS1 and its closest cultured and uncultured relatives were constructed with MEGA X (Kumar et al., 2018) based on the Maximum-likelihood method and Tamura-Nei model with 1,000 bootstrap replications after multiple alignments using MUSCLE (Edgar, 2004). In addition to 16S rRNA gene analyses, the purity of enrichment cultures and putative isolates of strain KaireiS1 was routinely checked by performing fluorescence in situ hybridization (FISH) with the cy-3 labeled oligonucleotide probe 660 (DBB660: 5'-GAA TTC CAC TTT CCC CTC TG -3'; Devereux et al., 1992). For this purpose, 1ml of the respective culture was diluted 1:10 in sterile PBS (phosphate buffered saline) and fixed with formaldehyde [4% (v/v)] for 2–4h at 22°C. Subsequently, the fixed cells were concentrated on polycarbonate filters (type: Nuclepore, 0.2μm pore size, Whatman, Buckinghamshire, United Kingdom), washed with sterile PBS, and stored at −20°C until further usage. The hybridization with probe DBB660 (final concentration 50ng/μl) was performed at 60% formamide as described in Glöckner et al. (1999). Counter-staining of all cells was performed using diamidino-2-phenylindole (DAPI) at a final concentration of 5μg/ml. All fluorescence signals on filter sections were inspected using an AxioImager M2 microscope (Carl Zeiss, Oberkochen, Germany). Additionally, the putative isolates of strain KaireiS1 were subjected to Gram staining (Gram, 1884) and Transmission electron microscopy (TEM, as described in Keuter et al., 2011), using glutaraldehyde-fixed cells and a LEO 906E electron microscope (Carl Zeiss). For purity checks at the end of hydrogen and sulfate consumption and CO_2_-incorporation experiments, the 16S rRNA gene was PCR-amplified as described above. Instead of the construction and sequencing of clone libraries however, the purified PCR product was directly sequenced.

### Growth With Inorganic Substrates

To test for the ability of KaireiS1 to grow under autotrophic conditions on hydrogen oxidation with Fe(III) as electron acceptor, MJ medium amended with 20mM Na_2_MoO_4_ was inoculated with a culture that was actively growing on hydrogen with sulfate as electron acceptor. Na_2_MoO_4_ is an efficient inhibitor of sulfate reduction (Oremland and Capone, 1988; Stoeva and Coates, 2019) and was added to prevent production of H_2_S by sulfate reduction as sulfate was still present in the medium. To avoid abiotic Fe(III) reduction by H_2_S that was contained in the inoculum, the culture was flushed with H_2_:CO_2_ (80:20) for >1h before Fe(III) (5mM) was added. By this procedure, the concentration of H_2_S from the inoculum was minimized to *ca.* 6μM, such that a maximum of *ca.* 50μM of Fe(III) reduction in the later incubation could be attributed to abiotic Fe(III) reduction. For determining the H_2_S concentration in the inoculated culture, sampling and colorimetric analysis *via* the Cline assay (Cline, 1969) was done as described below for the sulfate reduction rate measurements. Fe(III) was added as ferrihydrite that was synthesized as described by Schwertmann and Cornell (2008). For determination of Fe(II) and Fe(III) concentrations over time during the incubation at several timepoints, 500μl of the culture were sampled with a N_2_-flushed syringe and immediately added to 100μl of 6M HCl (1M HCl final concentration) for acid Fe extraction. After 1h, the Fe extraction was stopped by centrifugation (5min, 20,000 x *g*) and the supernatant was sampled and stored at 4°C in the dark until analysis of Fe concentrations. Fe(II) and total Fe concentrations in the extracts were determined spectrophotometrically with the ferrozine assay (Stookey, 1970) performed on 96-well plate scale with a microplate reader (SPECTROstar Nano, BMG Labtech). For total Fe concentrations, all Fe(III) was reduced to Fe(II) with hydroxylamine hydrochloride before the assay. Fe(III) was calculated from the difference between Fe(II) and total Fe concentrations. To test for the enhancement of Fe(III) reduction capability by acetate, the same incubations were performed also with the additional amendment of 100μM acetate (Holmes et al., 2004).

KaireiS1’s ability to use sulfate as electron acceptor was tested by cultivating KaireiS1 in MJ medium under an H_2_:CO_2_:He atmosphere (2:20:78) and in the absence of any organics. Sulfate reduction of the cultures and cell-free controls was monitored by the quantification of sulfate and sulfide in the culture medium. Samples for quantification of both sulfate and sulfide concentrations in the cultures were taken with a N_2_-flushed syringe. For sulfide quantification, 500μl of the culture were immediately added to 500μl of 5% (w/v) zinc acetate to fix sulfide as zinc sulfide and stored at 4°C until analysis. Sulfide concentrations were determined spectrophotometrically with the Cline assay (Cline, 1969) performed on 96-well plate scale with a microplate reader (SPECTROstar Nano, BMG Labtech). For sulfate quantification, 500μl of the culture were added to a reaction vial and sparged with CO_2_ for 1min to remove all sulfides. Afterward, sulfate samples were centrifuged for 15min at 20,000 x *g*, and the supernatant transferred into fresh tubes and stored at 4°C until analysis. Sulfate concentrations were measured on 1:50 diluted samples by ion chromatography (METROHM 761 Compact, equipped with a Metrosepp A Supp5-250/4.0 column) using IAPSO as standard. At each time point, a subsample (0.5 or 1ml) was taken, fixed, and concentrated on polycarbonate filters (0.22μm, type Nuclepore) as described above for cell enumeration and purity checks. The DAPI-stained cells were counted from filter sections, and FISH was performed with filter sections from the last time point. Additionally, at the end of the experiments, a subsample of each culture was taken and frozen at −70°C for 16S rRNA gene analyses (as described above). All analyses were performed in triplicates.

For CO_2_-incorporation experiments, NaHCO_3_-free MJ medium was distributed in serum bottles and flushed with N_2_ for 5min. Subsequently, pure hydrogen was added to a final concentration of 2% (v/v) in the headspace. Prior to inoculation, pre-cultures of KaireiS1 were flushed with N_2_ for 5min. The cultures (and controls containing only the medium) were supplemented with ^14^C-labeled NaHCO_3_ (specific activity of 50–60mCi/mmol; Hartmann Analytic, Braunschweig, Germany) to a final activity of 37kBq/ml. Non-labeled controls (for cell counting and purity checks) were inoculated from the same pre-cultures and supplemented with 16μM NaHCO_3_. After 24h, cell growth of the ^14^C-labeled cultures was stopped by adding formaldehyde to a final concentration of 4% (v/v) with an incubation time of 2h. Subsequently, 15ml of the fixed cultures and medium controls were concentrated on polycarbonate filters (Nuclepore type, 0.22μm pore size) and washed with sterile PBS buffer. Unincorporated CO_2_ was removed from the filters by incubating them for 2h in a desiccator containing 1M HCl. The filters (as well as untreated filters as blanks) were mixed with 5ml of Scintillation cocktail (Ultima Gold, PerkinElmer, Waltham, MA, United States) each and incubated in the dark overnight. The activity of the filters was measured with a TriCarb Scintillation Counter (PerkinElmer). CO_2_-fixation rates were calculated as previously described (Perner et al., 2010) based on a DIC content of 1.2mM resulting from carry-over effects of the pre-cultures. CO_2_-fixation rates of the medium controls (which were in the range of the blanks) were used for the correction of KaireiS1’s rates. For cell counting and purity checks (*via* FISH), 1ml of the non-labeled cultures was sampled each at t0 and t1 (24h), fixed with formaldehyde and concentrated on polycarbonate filters as described above. Additionally, a subsample of 1ml was taken and frozen at −70°C for 16S rRNA gene analyses (as described above). All analyses were performed in triplicates.

The optimal salinity for KaireiS1’s growth in the absence of organics [under an H_2_:CO_2_ (80:20) atmosphere] was tested by adding different amounts of NaCl into modified MJ medium (containing 2g/l NaCl). The tested NaCl concentrations ranged from 2 to 40g/l and all of the cultures were incubated at 28°C. Cell growth and purity was monitored in all cases *via* FISH as described above.

### Growth With Organic Substrates

For growth tests with different organic substrates, a modified basal medium (Widdel and Pfennig, 1981) with the addition of 34mM NaCl, 15mM MgCl_2_·6H_2_O, and trace element solution SL-10 (DSMZ medium 320) was autoclaved and purged with N_2_:CO_2_ (80:20) gas mixture (Westfalen AG). Vitamin solution (DSMZ medium 141) was added to the cooled medium and the pH was adjusted to 7.0 with 5% Na_2_CO_3_ under the same gas atmosphere. The following compounds were added individually into basal medium containing 30g/l of NaCl: alkanes (hexane, heptane, octane, decane, and pentadecane), each 20μl per 50ml medium; sugars (glucose and fructose), 20mM each; sodium propionate, 31mM; lactate, 20mM; pyruvate, 20mM; fumarate, 20mM; formate, 20mM; glutamate, 20mM. The headspace was filled with N_2_:CO_2_ (80:20) gas mixture each.

To test whether KaireiS1 is able to grow with hydrogen as electron donor in the presence of acetate, 37mM sodium acetate was added to the basal medium containing NaCl (30g/l) and the headspace was filled with H_2_:CO_2_ (80:20) gas mixture.

### Hydrogen Consumption and Enzyme Activity Measurements

For hydrogen consumption measurements, KaireiS1 was grown under autotrophic conditions in MJ medium under an H_2_:CO_2_:He (2:20:78) atmosphere with sulfate as electron acceptor. Hydrogen consumption measurements and calculation of hydrogen consumption rates of strain KaireiS1 and controls (sterile MJ medium) were performed as described by Hansen and Perner (2015) with a Trace GC Ultra gas chromatograph (ThermoFisher Scientific, Waltham, MA, United States), using a ShinCarbon ST 100/120 column (Restek Corporation, Bellefonte, PA, United States) and a Pulsed Discharge Detector (Vici Valco Instruments, Houston, TX, United States). For cell counting, 0.5 or 1ml of the cultures were sampled at each time point and fixed as described above. DAPI-stained cells were counted from filter sections and at the end of the experiment, and a routine purity check with FISH was performed. Additionally, subsamples from the last time point were frozen at −70°C and used for 16S rRNA gene analyses (as described above). All experiments (including controls) were performed in triplicates.

For hydrogen uptake (hydrogenase) and carbon monoxide dehydrogenase (CODH) activity measurements, the cultures of KaireiS1 were grown autotrophically in MJ medium under H_2_:CO_2_ (80:20) atmosphere for 10–14days. Partially purified protein extracts for spectrophotometric hydrogenase assays were prepared and hydrogen uptake activity measurements were performed using methylviologen as artificial electron acceptor, as previously described (Adam and Perner, 2018b). Briefly, cells were harvested and washed under anoxic conditions, lysed *via* sonication and (after the removal of cell debris) membrane and soluble fractions of the protein extracts were separated by ultracentrifugation at 100,000 x *g* for 1h. The specific hydrogen uptake activity of both fractions was measured at 28°C.

Crude cell extracts for CODH activity measurements were prepared under a 100% N_2_-atmosphere inside an Inert Glovebox (SylaTech GmbH, Walzbachtal, Germany). Cells from a total culture volume of 500ml were harvested by centrifugation (10,000 x *g*, 20min, 8°C) and washed with sucrose washing buffer (pH 7.8) containing 100mM Tris HCl, 1mM EDTA, 0.2g sucrose/ml, and 1mM DTT. Cell pellets were resuspended in 1ml CODH assay buffer [100mM Tris–HCl, 1mM EDTA, 10mM MgCl_2_ (pH 7.8), and 1mM DTT] containing 5mg/ml Lysozyme. Cell lysis was performed at 37°C and stopped after 2h by a final centrifugation (10,500 x *g*, 20min, room temperature). Supernatants were stored in Hungate Tubes under an N_2_ atmosphere and were used for analyzing the specific activity of CODH. Protein concentrations of the crude cell extracts were determined according to Bradford (1976). CODH activity assays were performed spectrophotometrically using oxidized methylviologen as an electron acceptor. All measurements were performed at 28 and 55°C under anaerobic conditions. Therefore, an UV/Vis crystal cuvette was constantly flushed with N_2_ and filled with anoxic CODH assay buffer as well as 50mM methylviologen. Subsequently, about 50μl of 5mM sodium dithionite were added until the mixture turned lightly blue and the prepared crude cell extracts were then added to the reaction mixture. The enzymatic reaction of the control was observed spectrophotometrically at 602nm (V-630 UV/VIS Spectrophotometer with EHCS-760 Peltier Cell Holder, Jasco, Groß-Umstadt, Germany) under an N_2_ atmosphere. Carbon monoxide oxidation was stimulated by flushing the cuvette with 100% CO gas for 30s and the reaction was likewise observed spectrophotometrically over a period of 10min. Mean values of CODH activities were calculated from three replicates based on an extinction coefficient of 9,800l*mol^−1^*cm^−1^.

## Results

### Enrichment and Isolation of Strain KaireiS1

The initial enrichment culture from an inactive chimney fragment from the Kairei vent field *via* serial transfers into MJ medium (without organics) with hydrogen as electron donor was characterized by the formation of black precipitates and a sulfidic smell. The 16S rRNA gene analysis of the enrichment revealed a community of five bacterial species with a deltaproteobacterial representative as the dominant species (72%). The other species were affiliated with the *Sunxiuqinia* genus (15%), *Pseudomonas* (8%), *Spirochaeta* (<5%), and the *Dokdonia* genus (<5%). Repeated enrichments and dilutions under different environmental conditions (i.e., with or without organics) selected for a deltaproteobacterial strain of the Desulfobulbaceae family, namely KaireiS1, in hydrogen-amended, autotrophically grown cultures. In these cultures, no other bacterial species were detectable *via* FISH or 16S rRNA gene analyses of clone libraries. Hydrogen-free cultures supplemented with glucose, fructose or pyruvate and fumarate, however, demonstrated a mix of KaireiS1 and *Sunxiuqinia* sp., where *Sunxiuqinia* accounted for 18% (fructose), 54% (pyruvate and fumarate), and 72% (glucose) of the total cell mass, respectively (Table 1). Such a mix also developed when an autotrophically grown, apparently pure culture of KaireiS1 was transferred into medium amended with the respective organic compounds. Generally, cultures of strain KaireiS1 grown with organic substrates showed a decrease in cell counts of KaireiS1 (compared to autotrophically grown cultures), with glucose as the only exception (Table 1). No growth at all was detected when glutamate, fumarate or the short-chain alkanes hexane, heptane, octane, and decane were supplied. The addition of pentadecane however, led to a slight growth of strain KaireiS1. Higher growth rates were achieved with lactate and propionate as electron donors, coupled to the reduction of sulfate (Tables 1 and 2).

**Table 1:** Cell counts of the cultures growing with different substrates.

Substrate	Cell numbers (cells/ml)		Percentage of strain KaireiS1 cells (%)
	FISH	DAPI	
Propionate	1.24E+06±1.27E+05	1.23E+06±1.16E+05	101±2
Lactate	1.19E+06±5.38E+04	1.13E+06±1.39E+05	106±10
Propionate+lactate	1.05E+06±9.61E+04	8.87E+05±3.73E+04	119±7
Pyruvate+fumarate	1.77E+06±7.08E+05	3.85E+06±9.18E+05	46±10
Formate	6.59E+05±3.38E+04	7.30E+05±7.00E+04	91±5
H_2_	2.83E+06±5.05E+05	2.89E+06±6.51E+05	99±6
H_2_+acetate	1.82E+06±1.29E+06	1.83E+06±1.33E+06	101±5
Pentadecane	9.13E+05±1.16E+05	9.89E+05±2.29E+05	94±14
Glucose	2.66E+06±4.85E+05	1.01E+07±2.70E+06	28±11
Fructose	7.02E+05±1.21E+05	8.57E+05±4.04E+04	82±10
Just after inoculation	6.44E+05±3.70E+04	6.49E+05±2.91E+04	99±5

The cell numbers were counted from FISH with DSB660 probe and DAPI staining.

**Table 2:** Characteristics of Strain KaireiS1 compared to those of three of the closest cultured relatives *Desulfobulbus mediterraneus* (Sass et al., 2002), *Desulfobulbus propionicus* (Widdel and Pfennig, 1982), and *Desulfobulbus elongatus* (Samain et al., 1984).

Characteristic	KaireiS1	*D. mediterraneus*	*D. propionicus*	*D. elongatus*
Source	Inactive chimney from hydrothermal deep-sea vent	Deep-sea sediment	Anoxic freshwater ditches and ponds	Anaerobic digester
Cell shape	Straight or slightly curved rod	Oval-shaped with rounded ends	Ellipsoidal to lemon-shaped	Straight or slightly curved rod
Cell size	Around 1.5–2.5μm long and 0.5μm wide	1.4–3.2μm long and 1.2–1.7μm wide	1.8–2.0μm long and 1.0–1.3μm wide	1.5–2.5μm long and 0.6–0.7μm wide
Motility	Motile, with flagella	Motile, with flagella	Immotile, no flagellum, surrounded by pili	Motile with a single polar flagellum
Salinity NaCl (g/L)	10–40g/l, optimal 30g/l	10–70g/l, optimal 20g/l	ND	ND
Compounds tested as electron donor and carbon source				
Propionate	+	+	+	+
Lactate	+	+	+	+
Propionate + lactate	+	ND	ND	ND
Pyruvate	−	+	+	+
Fumarate	−	+	−	−
Pyruvate+fumarate	+?	ND	ND	ND
Formate	(+)?	−	−	−
Glutamate	−	+	ND	ND
H_2_	+	−	−	−
H_2_+acetate	+	−	+	+
				
Hexane	−	ND	ND	ND
Heptane	−	ND	ND	ND
Octane	−	ND	ND	ND
Decane	−	ND	ND	ND
Pentadecane	+	ND	ND	ND
				
Glucose	+?	+	−	−
Fructose	(+)?	(+)	−	−

+, used; (+), weakly used; −, not used; ?, growth was detected but needs further clarification; and ND, not determined or no data available.

### Phylogeny and Morphology of Strain KaireiS1

**Figure 1:**
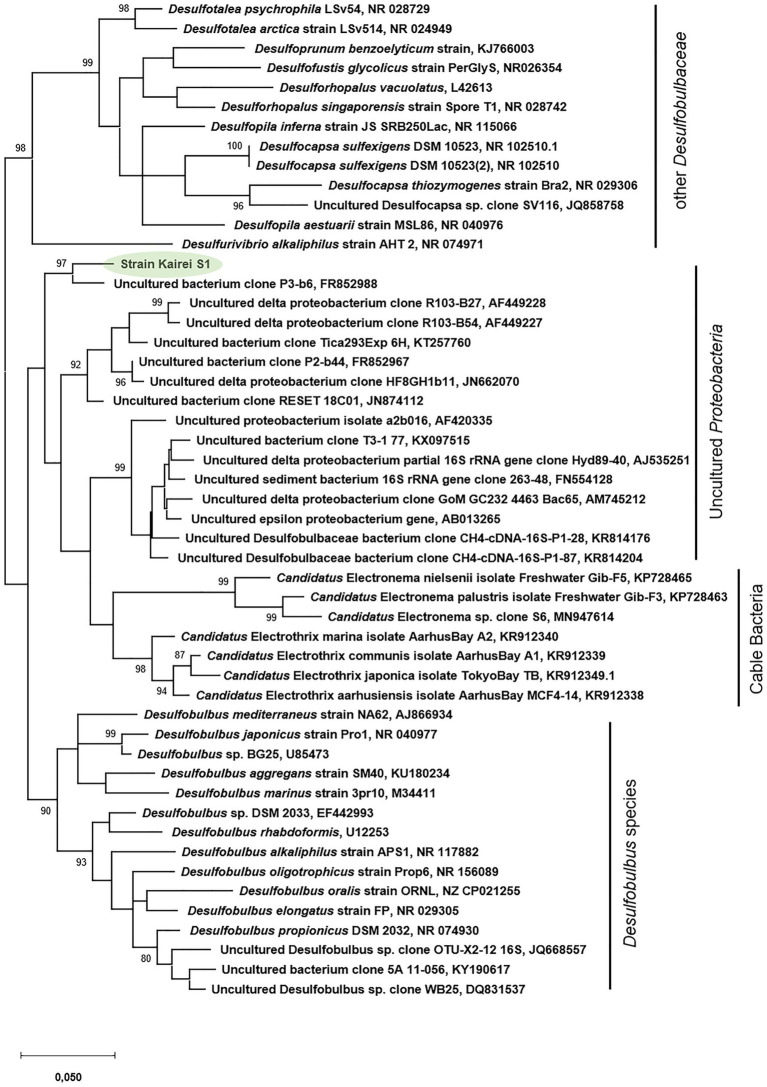
Phylogenetic relationship of 16S rRNA gene sequences of strain KaireiS1 and its closest relatives. Bootstrap values are only displayed if above 80%. The scale bar represents the expected number of changes per nucleotide position.

Phylogenetic analysis of the 16S rRNA gene (1,462bp, GenBank accession number: MH763813) of strain KaireiS1 placed it into the class of Deltaproteobacteria and clustered it among several uncultured deltaproteobacterial organisms (Figure 1). Based on a comparison of the 16S rRNA gene with entries in public databases, the closest cultured representatives are strains *Desulfobulbus* sp. DSM2033 (93.9% identity), *Desulfobulbus* sp. BG25 (93.7% identity), and the species *Desulfobulbus mediterraneus* (93.2% identity; Sass et al., 2002), *D. rhabdoformis* as well as *D. propionicus* (92.8% identity each).

**Figure 2:**
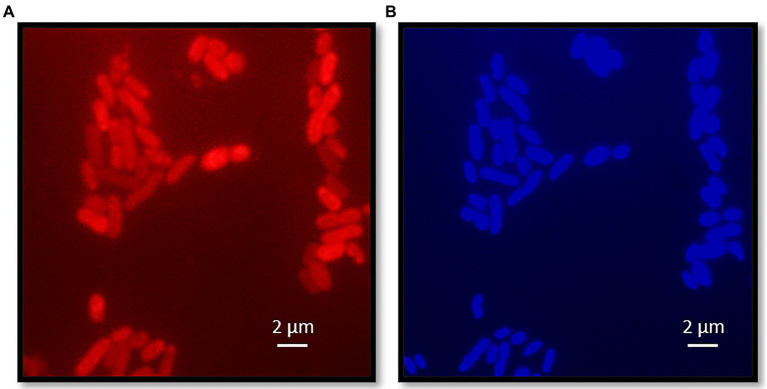
Fluorescence microscopy of strain KaireiS1. Strain KaireiS1 grew with hydrogen as electron donor and sulfate as electron acceptor. After fixation the cells were labeled with probe DBB660 *via* fluorescence *in-situ* hybridization (FISH; **A**) and stained with diamidino-2-phenylindole (DAPI; **B**).

**Figure 3:**
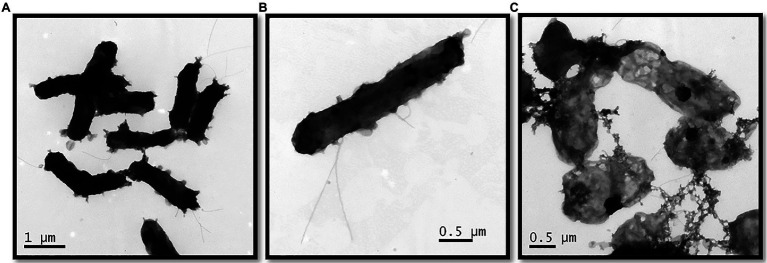
Transmission electron micrograph of strain KaireiS1 grown with hydrogen as electron donor and sulfate as electron acceptor in the mid-exponential growth phase **(A,B)** and stationary growth phase **(C)**.

Cells of strain KaireiS1 usually appear singly or in pairs and hybridize with the *Desulfobulbus* specific probe DBB660 (Figures 2, 3A). The cell shape appears to depend on the age of the cells (Figure 3). In the mid-exponential growth phase, cells were straight or slightly curved rods with round ends, around 1.5–2.5μm in length and around 0.5μm in width (Figures 3A,B). These are very similar to *Desulfobulbus elongatus* (Samain et al., 1984). In the stationary growth phase, cells were oval (Figure 3C). Cells of strain KaireiS1 are motile with several flagella at poles or on the sides and vesicles (visible as lighter shaded circular structures) can surround the cells (Figures 3A,B).

### Growth on Inorganic Substrates

**Figure 4:**
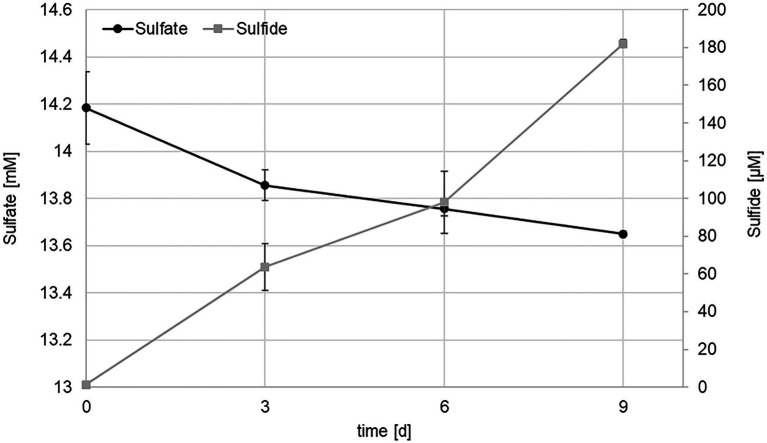
Sulfate (black) and sulfide (gray) concentrations in cultures of strain KaireiS1 grown in MJ medium under a H_2_:CO_2_:He (2:20:78) atmosphere. Error bars indicate the standard deviations from three independent experiments.

Routinely, strain KaireiS1 was grown in MJ medium (without organics) under an atmosphere of H_2_:CO_2_ (80:20) at 28°C. Growth was observed at salinities between 20 and 40g/l with the optimum at 30g/l, which corresponds to the standard salinity of MJ medium. The temperature optimum was 28°C but growth was observed at a range from 25 to 37°C. Inoculation with glycerol or DMSO cryo stocks (with varying concentrations) did not lead to actively growing cultures, making it necessary to transfer KaireiS1 every 4weeks. Actively growing cultures were characterized by the formation of black (iron sulfide) precipitates and a strong sulfidic smell, suggesting that KaireiS1 belongs to the group of sulfate reducing bacteria (SRB). To prove this hypothesis, sulfate and sulfide measurements were performed, applying the same growth conditions as used for hydrogen consumption measurements. Within 9days, the sulfate concentration decreased from 14.2mM to 13.6mM, while the sulfide concentration increased from 1.4μM to 182.0μM (Figure 4). The electrons used to reduce the sulfate under the applied standard growth conditions are derived from molecular hydrogen (for details see below).

Tests for the ability of KareiS1 to grow on Fe(III) as electron acceptor and molecular hydrogen as electron donor showed that Fe(III) was initially relatively quickly reduced to Fe(II) (ca. 3mM within 14days), but the Fe(III) reduction rates decreased by about an order of magnitude when the culture was transferred to fresh medium (10% inoculum; *ca.* 200μM reduced within 14days, Supplementary Figure S1). This indicates that it cannot be continuously cultivated under these conditions. *Desulfobulbus propionicus* can be continuously cultured with H_2_ and Fe(III) if 100μM of acetate is added (Holmes et al., 2004). However, with the addition of 100μM acetate, Fe(III) reduction rates after first inoculation and transfer into fresh medium did not differ considerably under the provided conditions from non-acetate amended KaireiS1 cultures, indicating that acetate does not increase the capability of KaireiS1 to grow on H_2_ and Fe(III) (Supplementary Figure S1).

In contrast to its nearest cultured relatives of the *Desulfobulbus* genus and many other *Desulfobulbaceae* members, strain KaireiS1 appears to grow autotrophically, demonstrated by repeated inoculations without the addition of organics (standard conditions). Furthermore, the assimilation of ^14^C-labeled CO_2_ was monitored, resulting in a CO_2_-fixation rate of 0.21±0.08nmol CO_2_*ml^−1^*h^−1^, or 0.19±0.08 fmol CO_2_*cell^−1^*h^−1^. FISH analyses of unlabeled control cultures (also used for cell counting) showed that at least 99% of the cells hybridized with the DBB660 probe (Supplementary Figure S2). Additional sequencing of the respective 16S rDNA resulted in clean sequences, completely matching KaireiS1’s 16S rRNA gene. To test whether KaireiS1 uses the reductive Acetyl-coA pathway (Wood-Ljungdahl pathway) for autotrophic CO_2_-fixation, which is widely distributed among autotrophic *Deltaproteobacteria*, we performed a specific enzyme assay targeting the key enzyme of the Wood-Ljungdahl pathway: carbon monoxide dehydrogenase (CODH). This enzyme assay, performed with crude extracts of KaireiS1, resulted in a specific CODH activity of 253nmol CO*min^−1^*mg^−1^ at 28°C. Measurements of CODH activity at 55°C revealed a nearly 20-fold higher CODH activity (4,850nmol CO*min^−1^*mg^−1^), which exceeds the expected increase related to thermodynamic effects based on the Arrhenius equation and Q10 coefficient (2–3 fold increase of enzyme activity per 10°C rise in temperature).

### Hydrogen Consumption and Hydrogen Uptake Activity

**Figure 5:**
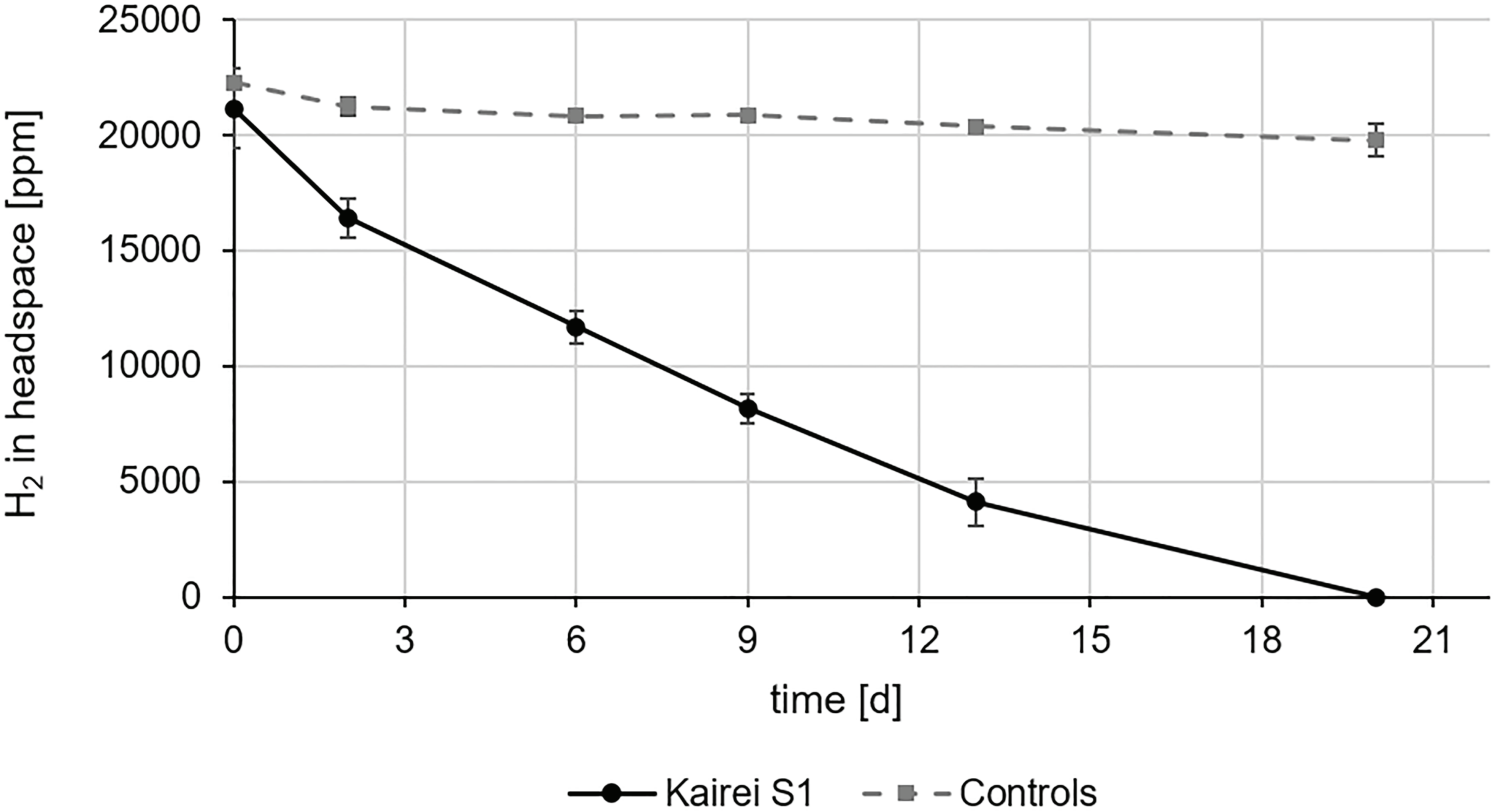
*In vivo* hydrogen consumption of strain KaireiS1 grown chemoautotrophically with hydrogen as electron donor and sulfate as electron acceptor. A control experiment was set up to exclude hydrogen leakage from the vessels. Error bars indicate the standard deviations from three independent experiments.

Strain KaireiS1’s hydrogenotrophy was confirmed by hydrogen consumption measurements without any additional organic or inorganic electron donors and with sulfate (contained in the MJ medium) as the main electron acceptor. Within 20days, a complete removal of hydrogen from the head space [H_2_:CO_2_:He (2:20:78)] of the cultures was achieved (Figure 5). As also observed in the sulfate consumption experiments, purity checks (FISH and 16S rRNA gene sequencing) at the last time point demonstrated an apparently pure culture of KaireiS1 (Supplementary Figure S2). Within the first 48h of incubation, the highest hydrogen consumption rate could be observed (10.2±1.8 fmol H_2_*h^−1^*cell^−1^), which is higher than some previously reported hydrogen consumption rates of pure cultures (Häring and Conrad, 1991; Klüber and Conrad, 1993; Hansen and Perner, 2016) and in the medium range of those of incubation experiments with hydrothermal vent samples (Adam and Perner, 2018a and references therein).

**Figure 6:**
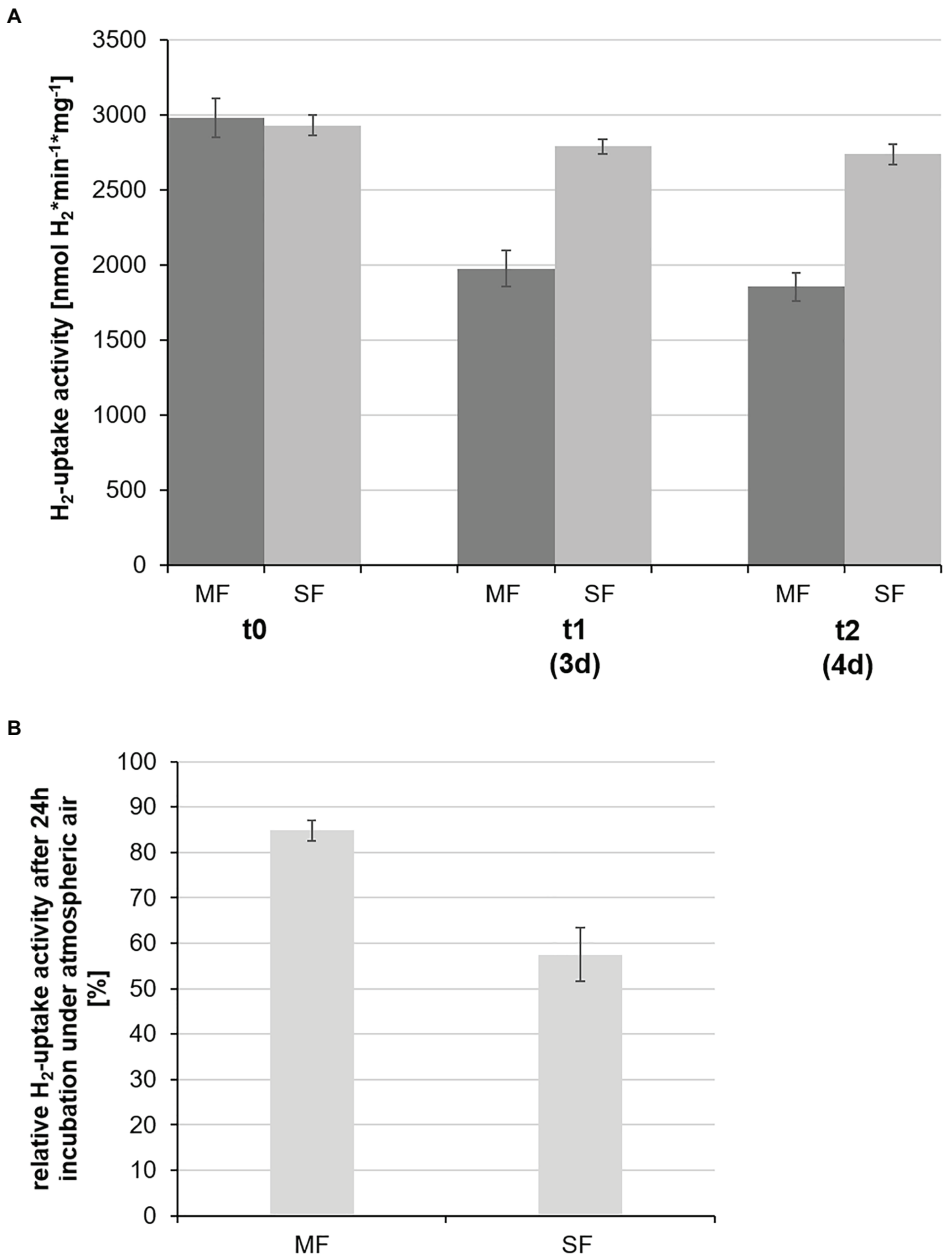
Specific hydrogen uptake activities of the soluble (SF) and membrane (MF) protein fractions of strain KaireiS1. The measurements at the different time points (0, 3, and 4days) were carried out using the same protein extracts, stored under oxygen-free conditions at 4°C **(A)**. The stability of the respective hydrogenases was also tested by incubating the protein extracts from day 3 for 24h under atmospheric air at 4°C with the subsequent determination of the relative hydrogen uptake activities at time point t_2_ **(B)**. Error bars indicate the standard deviations from three independent experiments.

For measurements of KaireiS1’s specific hydrogen uptake activity membrane and soluble fractions of cell extracts were separated by ultracentrifugation, allowing to identify the location of the respective hydrogen uptake active enzymes. Since hydrogen uptake activity was found in both fractions (Figure 6), at least two uptake hydrogenases must be active in strain KaireiS1 during chemoautotrophic growth with hydrogen as the only energy source. At the initial measurement, both hydrogen uptake activities were nearly identical (Figure 6A) with 2,981±129nmol H_2_*min^−1^*mg^−1^ (membrane fraction) and 2,931±68nmol H_2_*min^−1^*mg^−1^ (soluble fraction). However, the temporal stability of the hydrogenases differed considerably: while the cytoplasmic hydrogenase still exhibited 95% of the initial activity after 3days of incubation at 4°C under anaerobic conditions, only 66% of the initial activity was observed for the membrane fraction under the same conditions (Figure 6A). The oxygen sensitivity of both protein fractions was tested by incubating the fractions at 4°C under atmospheric air for 24h (starting after the 3days incubation under anaerobic conditions). This test revealed that, albeit the temporal stability was less pronounced, the hydrogenase of the membrane fraction appears to be less sensitive to the exposure with oxygen. After the treatment with atmospheric air, 85% of the previously measured activity were observed for the membrane fraction, while only 56% of the soluble fraction’s activity remained (Figure 6B).

## Discussion

### Physiological and Morphological Traits of Strain KaireiS1

Commonly, deltaproteobacterial isolates from hydrothermal vent systems that can grow autotrophically with hydrogen as (the sole) electron donor are described as thermophiles with temperature optima between 50 and 61°C (Adam and Perner, 2018a and references therein). Strain KaireiS1 appears to be a currently rare exception to this phenomenon with its optimum at 28°C. This likely results from the isolation source being an inactive chimney in an active venting zone, with accordingly lower temperatures than those observed at actively fluid emitting chimneys. Furthermore, the temperature range of KaireiS1 corresponds to those of KaireiS1’s closest cultured relatives of the *Desulfobulbus* genus and representatives of the Cable Bacteria group (Lien et al., 1998; Sass et al., 2002; Müller et al., 2020).

#### Inorganic Substrates and Autotrophy

Although a few organic compounds can serve as energy source, the highest cell counts and thus the best growth of KaireiS1 is achieved with molecular hydrogen as energy source (Table 1). KaireiS1’s hydrogenotrophy was demonstrated by *in vivo* hydrogen consumption measurements as well as hydrogenase activity assays of partially purified cell extracts. As constrained by the detection of hydrogenase activity in both the membrane and the soluble fractions of the cell extracts, KaireiS1 harbors more than one active hydrogenase. The presence of multiple hydrogenases is widespread among sulfate-reducing *Deltaproteobacteria*, encompassing both [NiFe]- and [FeFe]-hydrogenases with hydrogen uptake, electron-bifurcation and hydrogen evolution functions (Greening et al., 2016; Baffert et al., 2019). Albeit, until recently, no membrane-associated hydrogenases were known among the closely related Cable Bacteria and hydrogen oxidation appeared to be an implausible way of energy conservation for this group (Kjeldsen et al., 2019; Müller et al., 2020). However, an experimental confirmation of the physiological roles of the (meta-)genome-derived hydrogenases and the putative hydrogenotrophy of Cable Bacteria is still pending (Müller et al., 2020). Especially in dynamic, hydrogen-rich habitats, such as hydrothermal vent systems, the use of several hydrogenases with putatively differing hydrogen affinities can provide an advantage for the conservation of 4, supporting primary biomass production. Previously reported hydrogen uptake activities of sulfate-reducing *Deltaproteobacteria* range among the highest microbial hydrogenase activities, exceeding those of strain KaireiS1 by far: e.g., 28μmol H_2_*min^−1^*mg^−1^ protein of *Desulfovibrio gigas* or 50μmol H_2_*min^−1^*mg^−1^ protein of *Desulfobulbus rhabdoformis* (Bell et al., 1978; Lien et al., 1998; Baffert et al., 2019). Due to diverging purification levels of the proteins and different assay types, however, such activities are often difficult to compare. Hydrogen uptake activities of different Proteobacteria, determined under comparable conditions as those of the here described KaireiS1 protein extracts, often do not considerably exceed 1μmol H_2_*min^−1^*mg^−1^ (Hansen and Perner, 2016; Adam and Perner, 2018b and references therein). Thus, strain KaireiS1’s hydrogen uptake activity appears to integrate into the range of the highly active hydrogenases of the *Deltaproteobacteria*.

**Figure 7:**
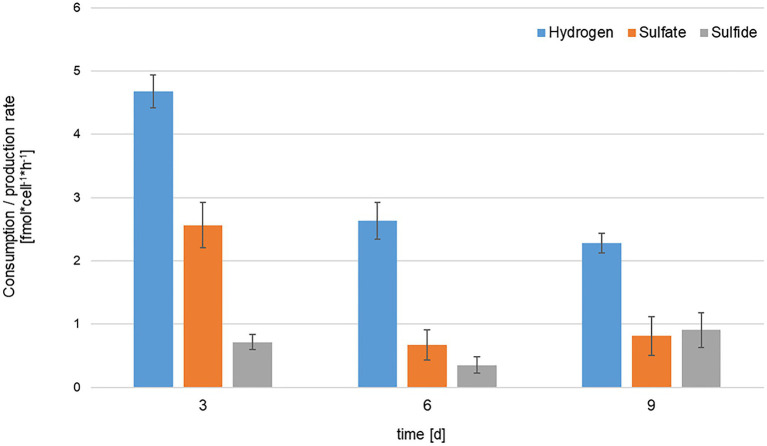
Comparison of hydrogen consumption (blue), sulfate consumption (orange) and sulfide production (gray) rates of KaireiS1 at three different time points. Rates are normalized to consumption/production per cell per hour and error bars indicate the standard deviations from three independent experiments.

Given the apparent and experimentally confirmed use of sulfate as electron acceptor, KaireiS1 belongs to the group of deltaproteobacterial SRB, which also constitute the largest phylogenetic group among the known SRB (Muyzer and Stams, 2008; Rabus et al., 2015). Since 4mol of hydrogen are required to reduce 1mol of sulfate, a ratio of 4:1 between hydrogen and sulfate consumption was theoretically expected (Konhauser, 2006). The comparison of the data for three different time points (normalized to fmol*cell^−1^*h^−1^; Figure 7), results in ratios between 1.82:1 (after 3days) and 3.95:1 (after 6days). The observed excess of sulfate consumption may result from the intracellular accumulation of sulfate or assimilatory sulfate reduction. However, the accumulation of sulfate was shown to be inhibited in marine SRB in the absence of sulfate limitation (Warthmann and Cypionka, 1990). Thus, assimilatory sulfate reduction, which is ubiquitously distributed among microorganisms and plants (Schiff and Fankhauser, 1981; Kredich, 2008), appears to be the most plausible reason for the discrepancy between the expected and actual ratios. Initially, the ratios between sulfate consumption and the formation of sulfide did not show the theoretically expected 1:1 proportion. However, a clear decrease over the three analyzed time points, ranging from 3.58 (after 3days) to 0.9 (after 9days) was observed (Figure 7). This indicates a delay in the formation of sulfide, which may be caused by the complex reduction process, involving the formation of a bisulfite intermediate (Rabus et al., 2015). Due to the volatile nature of sulfide, a slight bias caused by the sampling procedure may also be present.

Among the alternative electron acceptors used by *Desulfobulbus* species, Fe(III) represented a promising electron acceptor for KaireiS1 as its inactive chimney habitat displays a rich Fe-source dominated by chalcopyrite with a coating of Fe-oxyhydroxides resulting from seawater oxidation (Han et al., 2018). Furthermore, members of the Desulfobulbaceae family have been identified as potential Fe(III)-reducers in a sequence-based study (Reyes et al., 2016) and the oxidation of hydrogen coupled to Fe(III)-reduction would theoretically offer higher energy yields than if coupled to sulfate reduction (Adam and Perner, 2018a). However, a consistent growth of KaireiS1 with Fe(III) as the only electron acceptor could not be achieved, even when acetate was added to promote cell growth. Still, Fe(III) was significantly reduced upon the first inoculation into respective Fe(III)-containing media. As an abiotic reduction (caused by remaining sulfide of the pre-culture) can be excluded due to the experimental procedures, we propose that KaireiS1 is able to reduce Fe(III) but not able to conserve energy and grow from this reaction, as has previously been observed for two deltaproteobacterial *Desulfovibrio* species (Vandieken et al., 2006).

The energy conserved by hydrogen oxidation (coupled to sulfate reduction) serves the autotrophic biomass production of strain KaireiS1, demonstrated by a CO_2_-fixation rate that is in the upper range of CO_2_-fixation rates reported for hydrothermal fluid and sediment incubation experiments (Adam and Perner, 2018a and references therein; Böhnke et al., 2019). In this aspect, KaireiS1 shows greater resemblance to the closely related Cable Bacteria and vent-derived SRB outside of the Desulfobulbaceae family, than to its closest cultured (heterotrophic) relatives (cf. Table 2 and Rabus et al., 2015; Kjeldsen et al., 2019). The potential of Cable Bacteria to fix CO_2_ *via* the Wood-Ljungdahl pathway has been inferred from genomic analyses and confirmed for certain filaments of Cable Bacteria by means of CO_2_-incorporation experiments (Kjeldsen et al., 2019). Given the close relationship to Cable Bacteria, the Wood-Ljungdahl pathway appeared to be the most likely CO_2_-fixation way for KaireiS1. Indeed, we were able to measure a specific CODH activity, which is (based on measurements performed at the optimal growth temperature) up to 25 times higher than those of other deltaproteobacterial SRB, yet lower than that of *Desulfobacterium autotrophicum* or the acetogenic *Moorella thermoacetica*, the model organism of the Wood-Ljungdahl pathway (Ragsdale et al., 1983; Länge et al., 1989; Frederiksen and Finster, 2004; Gittel et al., 2010). Furthermore, KaireiS1’s CODH is characterized by a high thermal stability and an apparent temperature optimum that exceeds the growth temperature range. Taken together, the experimentally confirmed CO_2_-fixation ability and a CODH activity that is in the upper range of deltaproteobacterial SRB, strengthen our hypothesis that KaireiS1 is able to grow chemoautotrophically by fixing CO_2_ *via* the Wood-Ljungdahl pathway.

#### Use of Organic Substrates

Like most *Desulfobulbus* species, strain KaireiS1 can use propionate and lactate as electron donors, a trait that is missing in Cable Bacteria (e.g., Widdel and Pfennig, 1982; Sass et al., 2002; Kjeldsen et al., 2019). A putative fumarate respiration with pyruvate as electron donor (Kröger et al., 1992) is indicated by increased growth rates if pyruvate and fumarate are added simultaneously compared to the single addition of each substance (Tables 1 and 2). Among the tested alkane compounds, the growth of strain KaireiS1 can be detected with supplement of pentadecane but not hexane, heptane, octane or decane (Table 2). Several sulfate-reducing bacteria are characterized by the ability to oxidize *n*-alkanes under anaerobic conditions, e.g., *Desulfatibacillum* species, which were isolated from hydrocarbon-rich petroleum-contaminated marine sediments (So and Young, 1999; So et al., 2003; Cravo-Laureau et al., 2005). Furthermore, anaerobic oxidation of short-chain alkanes in hydrothermal sediments has also been reported to be coupled with sulfate reduction and play an important role in sulfur-cycling (Adams et al., 2013). The ability of strain KaireiS1 to metabolize the long-chain alkane pentadecane might therefore pose an advantage in adapting to diverse ecosystems. Whether members of the *Desulfobulbus* genus or Cable Bacteria share the ability to oxidize alkanes remains unknown and needs to be elucidated in the future, especially for those *Desulfobulbus* species isolated from oil fields.

#### Morphological Features

Morphological plasticity during different growth phases as observed for strain KaireiS1 can be found in various bacteria, e.g., as a response to changing environmental conditions, facilitating cell adhesion to surfaces or during biofilm formation (Young, 2006). Also, the formation of vesicles is a common phenomenon, which may serve horizontal gene transfer, detoxification, survival under stress conditions, interspecies interactions and promoting biofilm formation or pathogenity (Schwechheimer and Kuehn, 2015; Toyofuku et al., 2019). In the case of strain KaireiS1, the detoxification of sulfide through the vesicle structures would be a plausible hypothesis. However, based on experiments with a *Desulfovibrio* species, it is assumed that sulfide leaves bacterial cells by diffusion (Cypionka, 1987; Marietou et al., 2018). Based on our experiments, the role of KaireiS1’s vesicles cannot be predicted. Yet, the presence of morphological plasticity and membrane vesicles together may indicate the potential for the formation of biofilms, even though they have not been observed under the provided culturing conditions.

### Putative Co-culture With *Sunxiuqinia* Sp.

During the routine purity test from strain KaireiS1, 16S rRNA gene sequences of *Sunxiuqinia* species were detected when pyruvate+fumarate, glucose or fructose were separately added to the medium in which strain KaireiS1 was grown, accounting for 10–70% in these cultures (Table 1). Although several dilutions were performed, the 16S rRNA gene of *Sunxiuqinia* species was still detectable in the organic amended *Desulfobulbus* enrichments. The genus *Sunxiuqinia* is classified as a member of the *Bacteroidetes*, comprising heterotrophic, aerobic, or facultative aerobic species isolated from coastal marine sediments and deep sub-seafloor sediments (Qu et al., 2011; Chang et al., 2013; Takai et al., 2013; Yoon and Kasai, 2014). Among the *Desulfobulbaceae* family, a co-culture has been reported of the host-associated *Desulfobulbus oralis* and *Fusobacterium nucleatum*. Certain metabolites from *F. nucleatum* may be necessary for the growth of *D. oralis*, but the critical component provided to *D. oralis* by *F. nucleatum* is still unknown (Cross et al., 2018). In our work, *Sunxiuqinia* sp. might be required for strain KaireiS1 under the above described conditions. More likely however, *Sunxiuqinia* sp. might be more opportunistic, resulting in a boost of *Sunxiuqinia* sp.’s growth as a response to the addition of organic compounds. Under our experimental conditions, no physical interactions between strain KaireiS1 and *Sunxiuqinia* sp. could be detected and their possible relationship needs to be further investigated.

### Environmental Context

Strain KaireiS1 was enriched from a copper rich, chalcopyrite-dominated chimney fragment from the Kairei vent field. It was retrieved from the active zone of the vent field, yet our chimney sample showed no signs of vigorous venting. Although an accurate dating is not possible, the oxidation state of the chimney fragment indicates that the fluid flow ceased at the most 12months prior to our sampling campaign. Corresponding to this rather short period of inactivity, in beta diversity analyses, this chimney sample clustered with other inactive chimney samples from the Kairei field as well as those from actively venting sites, still indicating beneficial growth conditions for vent-associated microorganisms (Han et al., 2018). The microbial diversity of other inactive chimneys sampled from the Kairei vent field was also found to be higher than that typically observed at inactive chimneys and was related to the fact that Kairei belongs to the still nascent venting sites (Suzuki et al., 2004). Hence, the inactive Kairei chimneys still represent livable habitats for autotrophic (vent-associated) microorganisms such as KaireiS1. Kairei S1’s hydrogenotrophy may be maintained in the inactive vent environment by biogenically produced hydrogen, produced as a waste product in fermentative processes. Such syntrophies have already been hypothesized for actively venting habitats (Topcuoglu et al., 2016; Adam and Perner, 2018a). Another hydrogen source may be a yet unrecognized diffuse fluid flow in the sampling area. However, it is also possible that KaireiS1 makes use of a heterotrophic lifestyle, relying on waste products of other microorganisms. Given the higher growth rates under autotrophy, the latter may represent a survival strategy under less favorable conditions.

FISH targeting conserved regions of 16S rRNA genes from *Desulfobulbus* species (*via* probe DBB660; Devereux et al., 1992) demonstrated fluorescence signals for KaireiS1 cells (Figure 2). However, phylogenetic analysis showed that strain KaireiS1 is phylogenetically positioned in another cluster than the previously published *Desulfobulbus* species that were isolated from marine sediments or urban and industrial water systems (Figure 1). Furthermore, these uncultured bacteria are more closely related to Cable Bacteria (Figure 1), which are supposed to form a monophyletic sister group to the *Desulfobulbus* genus within the family of Desulfobulbaceae (Trojan et al., 2016; Kjeldsen et al., 2019). Based on a similarity cutoff of 94% (genera-level), a number of currently uncultured members of the same genus as KaireiS1 can be identified *via* blast searches, which cluster with KaireiS1 in the phylogenetic tree (Figure 1). Among these sequences from uncultured Deltaproteobacteria, the majority were derived from hydrothermally influenced habitats (chimneys, sediments, and basalt blocks) or associated to vent-inhabiting animals located in the Pacific Ocean (e.g., Lopez-Garcia et al., 2002; Teske et al., 2002; Schauer et al., 2011; Gulmann et al., 2015; Dekas et al., 2016). This indicates that the cluster of strain KaireiS1 (which currently cannot be assigned to a distinct genus within the Desulfobulbaceae family) encompasses bacteria that are well adapted to the environmental conditions present in hydrothermal vent systems.

The enrichment of the mesophilic strain KaireiS1 with its closely related uncultured bacterial relatives shows the potential for autotrophic sulfate reduction at hydrothermal vents exhibiting moderate temperatures. This potential has long been overlooked but is certainly worth to be further investigated.

## Data Availability Statement

Sequencing data presented in this study was deposited in the repository of the National Center for Biotechnology and can be found under Genbank accession number MH763813.

## Author Contributions

MP planned the study. YH performed bacterial enrichments, salinity tests, tests with organic substrates, and sample preparation for TEM-microscopy. NA performed initial sampling and culturing of the chimney fragment, H_2_-uptake activity and sulfide measurements, and CO_2_-incorporation experiments and was responsible for H_2_-consumption experiments. KL-M supervised sulfate and sulfide measurements and performed Fe(III)-reduction tests. RB performed CODH activity measurements. NA and YH wrote the manuscript with the contributions of MP, KL-M, AS, and US-S and with the approval of all authors.

## Funding

This work was supported by the Federal Institute for Geosciences and Natural Resources (BGR), Hannover, Germany, in the framework of the German exploration program INDEX.

## Conflict of Interest

The authors declare that the research was conducted in the absence of any commercial or financial relationships that could be construed as a potential conflict of interest.

## Publisher’s Note

All claims expressed in this article are solely those of the authors and do not necessarily represent those of their affiliated organizations, or those of the publisher, the editors and the reviewers. Any product that may be evaluated in this article, or claim that may be made by its manufacturer, is not guaranteed or endorsed by the publisher.

## Footnotes

^1^ https://blast.ncbi.nlm.nih.gov/Blast.cgi
