# Supplementary material for: Deltaproteobacterium Strain KaireiS1, a Mesophilic, Hydrogen-Oxidizing and Sulfate-Reducing Bacterium From an Inactive Deep-Sea Hydrothermal Chimney
Source: Front Microbiol. 2021 Sep 22;12:686276. doi: 10.3389/fmicb.2021.686276 (PMC8494109; doi:10.3389/fmicb.2021.686276)
Supplement: Supplementary file 1 [file Data_Sheet_1.PDF]

Supplementary information for:

Deltaproteobacterium strain KaireiS1, a mesophilic, hydrogen-oxidizing and sulfate-reducing isolate from an inactive deep-sea hydrothermal chimney

Nicole Adam[ǂ], Yuchen Han[ǂ], Katja Laufer-Meiser, Rebecca Bährle, Ulrich Schwarz-Schampera, Axel Schippers and Mirjam Perner*

ǂ the authors contributed equally to this study and share first authorship

*Corresponding Author: Mirjam Perner. Mailing Address: GEOMAR Helmholtz Center for Ocean Research Kiel, Geomicrobiology, Wischhofstr. 1-3, 24148 Kiel, Germany. Tel: +49-43-600-2837, Fax: +49-43-600-2941, E-mail: mperner@geomar.de

This file contains:

Supplementary figures S1 and S2

Supplementary figure S1:

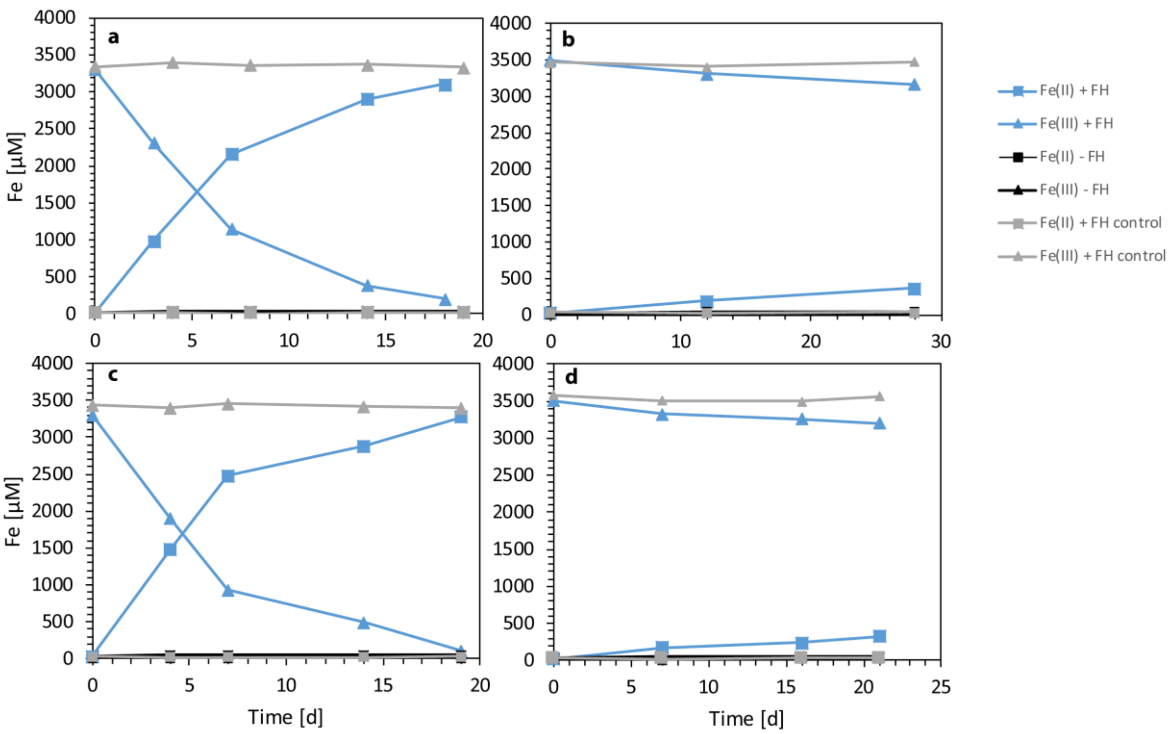

Fe(III) reduction tests. Fe(II)-concentrations (squares) and Fe(III)-concentrations (triangles) of different incubations and controls are displayed. Incubations of KaireiS1 amended with Fe(III) (ferrihydrite (FH)) are shown in blue, those without the amendment of FH are shown in black. Uninoculated controls amended with FH are displayed in grey. Panel a) shows the initial inoculation from pre-cultures grown with sulfate as electron acceptor (after bubbling to remove H_2S), while b) shows the first transfer of cultures grown with Fe(III) as electron acceptor (after bubbling to remove H_2S). Panels c) and d) display the initial inoculation and the first transfer with acetate amendment (after bubbling to remove H_2S), respectively.

Supplementary figure S2:

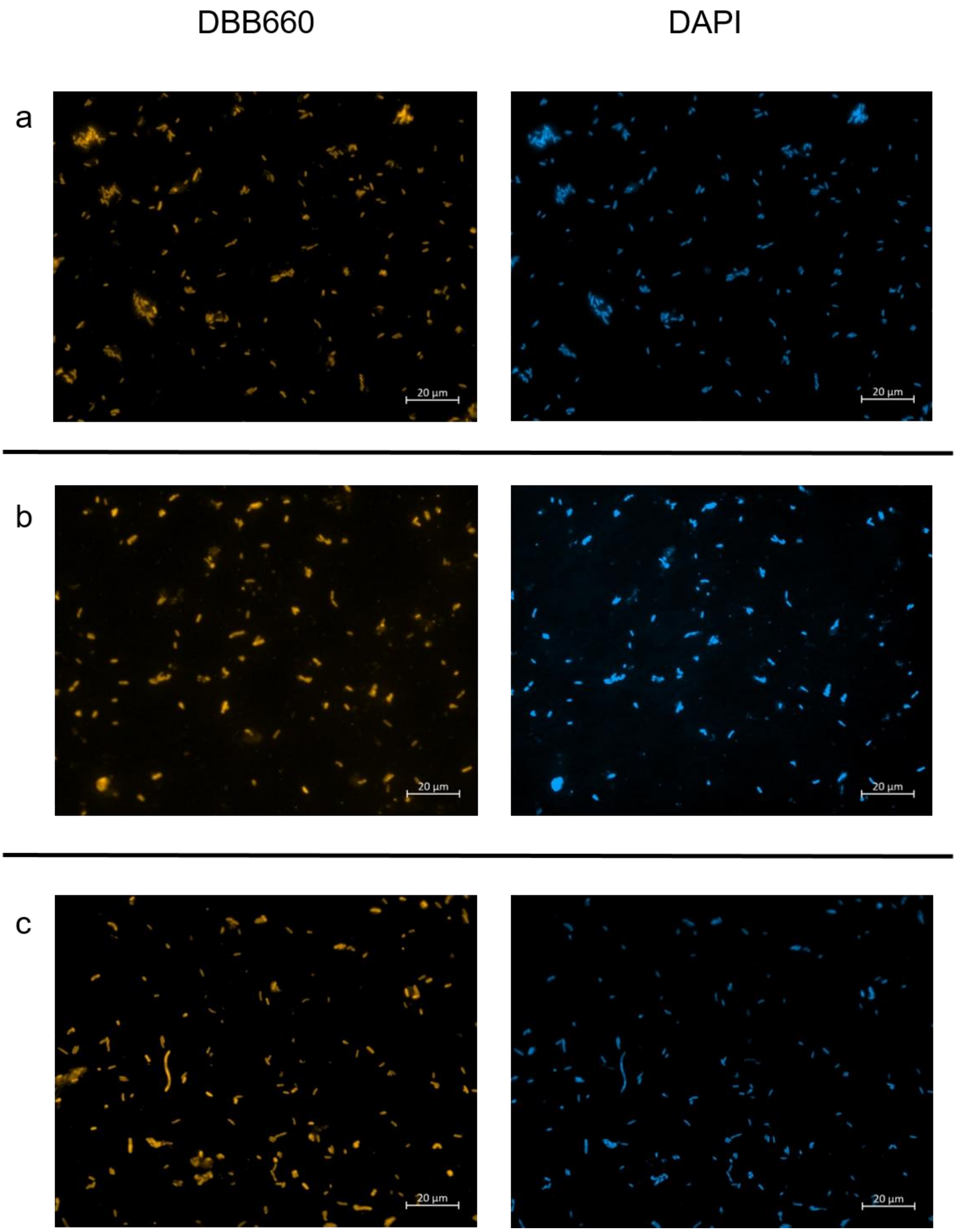

FISH microscopy of strain KaireiS1 at the end of substrate consumption experiments. Cells hybridized with the cy3-labeled probe DBB660 are marked in orange, DAPI-stained cells are marked in blue. Exemplarily, filter sections from the CO_2-incorporation experiment (a), sulfate consumption (b) and hydrogen consumption measurements (c) are shown.
